# Unravelling the Bi‐Functional Electrocatalytic Properties of {Mo_72_Fe_30_} Polyoxometalate Nanostructures for Overall Water Splitting Using Scanning Electrochemical Microscope and Electrochemical Gating Methods

**DOI:** 10.1002/advs.202401073

**Published:** 2024-04-12

**Authors:** Karthikeyan Krishnamoorthy, Parthiban Pazhamalai, Rajavarman Swaminathan, Vigneshwaran Mohan, Sang‐Jae Kim

**Affiliations:** ^1^ Nanomaterials & System Laboratory Major of Mechatronics Engineering Faculty of Applied Energy System Jeju National University Jeju 63243 South Korea; ^2^ Research Institute of New Energy Industry (RINEI) Jeju National University Jeju 63243 South Korea; ^3^ CSIR‐Advanced Materials and Processes Research Institute Bhopal Madhya Pradesh 462026 India; ^4^ Nanomaterials & System Lab Major of Mechanical System Engineering College of Engineering Jeju National University Jeju 63243 South Korea

**Keywords:** electrochemical gating, electrochemical water splitting, industrial alkaline electrolyzer, polyoxometalates, scanning electrochemical microscope (SECM)

## Abstract

This study reports the use of Keplerate‐type {Mo_72_Fe_30_} polyoxometalate (POMs) nanostructures as a bi‐functional‐electrocatalyst for HER and OER in an alkaline medium with a lower overpotential (135 mV for HER and 264 mV for OER), and excellent electrochemical stability. The bi‐functional catalytic properties of {Mo_72_Fe_30_} POM are studied using a scanning electrochemical microscope (SECM) via current mapping using substrate generation and tip collection mode. Furthermore, the bipolar nature of the {Mo_72_Fe_30_} POM nano‐electrocatalysts is studied using the electrochemical gating via simultaneous monitoring of the electrochemical (cell) and electrical ({Mo_72_Fe_30_} POM) signals. Next, a prototype water electrolyzer fabricated using {Mo_72_Fe_30_} POM electrocatalysts showed they can drive 10 mA cm^−2^ with a low cell voltage of 1.62 V in lab‐scale test conditions. Notably, the {Mo_72_Fe_30_} POM electrolyzers’ performance assessment based on recommended conditions for industrial aspects shows that they require a very low overpotential of 1.89 V to drive 500 mA cm^−2^, highlighting their promising candidature toward clean‐hydrogen production.

## Graphical abstract

A high‐performance bi‐functional {Mo_72_Fe_30_} POM electrocatalyst toward electrochemical water splitting is provided. Their bi‐functional electrocatalytic properties are studied using a scanning electrochemical microscope and electrochemical gating methods. The performance assessment of the {Mo_72_Fe_30_} POM water electrolyzer is tested under laboratory standard & industrially relevant conditions. Additionally, a self‐powered system for the production of green hydrogen is also demonstrated.

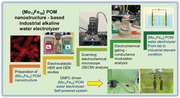

## 1 Introduction

Electrochemical water splitting is a cost‐effective route to produce hydrogen (a clean energy source) compared to the other traditional methods, attracting colossal interest as an alternate fuel due to the exponential increment in the global consumption of fossil fuels.^[^ ^1^, ^2^, ^3^ ^]^ The principle of electrochemical water splitting can be accompanied by two reactions: i) hydrogen evolution reaction (HER) and ii) oxygen evolution reaction (OER), which are both equally important for an adequate water splitting process.^[^ ^4^, ^5^ ^]^ The HER occurs at the cathode and converts the water into hydrogen gas, whereas oxygen gas is produced at the anode because of the OER.^[^ ^6^, ^7^ ^]^ HER is generally effective in acidic conditions (than base), whereas OER is effective only in basic conditions.^[^ ^8^ ^]^ Balancing these two reactions with appropriate catalysts might lead to better device performance for a water electrolyzer, on which the current research is focused during this decade.^[^ ^9^ ^]^ Even though noble platinum‐ and iridium oxide‐based catalysts were used as high‐performance HER and OER catalysts,^[^ ^10^, ^11^ ^]^ their high cost and complications in large‐scale manufacturing directly limit the commercialization of water electrolyzers.^[^ ^12^, ^13^ ^]^ Researchers have independently developed low‐cost catalytic materials for HER and OER during this decade.^[^ ^5^, ^14^ ^]^ However, using two different materials for anode and cathode leads to demerits such as cross‐contamination and higher manufacturing costs considering the commercialization aspects.^[^ ^15^, ^16^, ^17^ ^]^ Most of the better‐performing HER catalysts were reported to have poor OER activity and vice versa,^[^ ^18^, ^19^ ^]^ due to the significantly differential electrochemical reactions at the anode and cathode.^[^ ^3^ ^]^ Therefore, designing a universal catalyst that could provide better HER and OER activity in acidic/basic medium is extremely important considering the massive impact on the hydrogen economy.^[^ ^20^ ^]^ Recent studies demonstrated the bi‐functional catalytic properties of various nanostructured materials (single and multi‐component metals, metal oxides/hydroxides) with different morphology for both HER and OER applications.^[^ ^21^ ^]^ Further, the development of new synthetic strategies with tailored morphologies of bi/trimetallic materials from the transition metal dichalcogenides (TMDCs), metal phosphides, metal borides, M'Xenes and metal‐organic framework families showed superior electrocatalytic activity for overall water splitting.^[^ ^22^, ^23^, ^24^ ^]^ This led to an exponential increase in the overall quantity of research on developing water electrolyzers during the past five years, thus signifying the necessity of new and efficient bi‐functional electrocatalysts.^[^ ^25^ ^]^

In this context, polyoxometalates (POMs), a new class of nanosized transition metal oxide clusters in their highest oxidation states, are considered new candidates for electrochemical energy conversion and storage devices due to their structural merits, surface states, and excellent redox chemistry.^[^ ^26^, ^27^ ^]^ Typically, the structure of POMs comprises two or more transition metal oxyanions bonded with oxygen atoms in a 3D framework. The metal atom that forms the framework is termed an “addenda atom” (usually V or VI group transition metals).^[^ ^28^ ^]^ Being a subset of transition metal oxides (TMOs), POMs possess distinct physical and chemical properties compared to that of TMOs with their ability to form dynamic molecular structures such as a) Kegin, b) Waugh, c) Silverton, d) Lindqvist, e) Anderson, f) Dawson and g) Keplerate‐type structures.^[^ ^29^, ^30^, ^31^ ^]^ Among these, POMs with the structure of Keplerate are colossal TMO‐based nano‐capsules in that twelve pentagonal [(Mo)–Mo_5_O_21_(H_2_O)_6_]^6−^ units are connected via 30 linkers (such as molybdate, iron, chromium, and vanadate in mixed metal derivatives.^[^ ^27^ ^]^ In these, Keplerate‐type polyoxometalate {Mo_72_Fe_30_} clusters possess distinct properties such as visible‐light‐driven photocatalyst, an anode for Li‐ion batteries, the catalyst for organic synthesis, reversible redox mediators for the synthesis of metal nanoparticles, targeted drug delivery agent, and hybrid materials, proton conductivity, electrocatalyst for HER, and excellent guest/host chemistry.^[^ ^27^, ^31^, ^32^, ^33^, ^34^, ^35^ ^]^ To date, the electrocatalytic properties of {Mo_72_Fe_30_} POM for overall water splitting are not yet studied; however, it is expected that they can possess excellent electrocatalytic activity that is due to the presence of Mo and Fe atoms in their highest oxidation state.^[^ ^36^, ^37^ ^]^ In this work, we have demonstrated the better electrochemical catalytic properties of {Mo_72_Fe_30_} POM as a universal catalyst for HER and OER applications using standard electrochemical studies. Additionally, scanning electrochemical microscopic (SECM) analysis was used for understanding the relationship between the surface state of the {Mo_72_Fe_30_} POM, favoring electrochemical HER and OER. The bipolar nature of the {Mo_72_Fe_30_} POM was studied in detail with the aid of electrochemical gating analysis, which is one of the crucial techniques for understanding the electrochemical energy conversion and storage process in detail.^[^ ^38^, ^39^ ^]^ Further, a lab‐scale water electrolyzer is constructed to understand their device‐specific properties using {Mo_72_Fe_30_} POM as a bi‐functional catalyst, and a self‐powered system based on direct methanol fuel cell driven {Mo_72_Fe_30_} POM water electrolyzer is demonstrated.

## 2 Results and Discussion

### 2.1 Physico‐Chemical Characterization of {Mo_72_Fe_30_} POM Electrocatalyst

**Figure 1:**
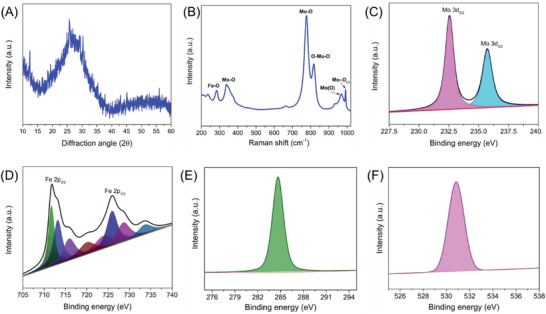
Physico‐chemical characterization of {Mo_72_Fe_30_} POM nanostructures. A) X‐ray diffraction pattern, B) laser Raman spectrum, C–F) Core‐level X‐ray photoelectron spectra of C) Mo 3d states, D) Fe 2p states, E) C 1s and F) O 1s states, respectively.

**Figure** **1**A displays the X‐ray diffraction pattern of {Mo_72_Fe_30_} POM nanostructures prepared via ultra‐sonication. The presence of a broad diffraction peak centered ≈26° indicated the amorphous nature of the prepared {Mo_72_Fe_30_} POM nanostructures. The observed diffraction pattern closely matches the previous report on wet chemically prepared {Mo_72_Fe_30_} POM nanostructures.^[^ ^31^ ^]^ The laser Raman spectrum of the {Mo_72_Fe_30_} POM nanostructures is provided in Figure 1B, indicates the presence of various vibrational bands related to Fe−O and Mo─O bonding in the prepared {Mo_72_Fe_30_} POM nanostructures. The sharp bands at 760 and 817 cm^−1^ are attributed to the Mo─O bonding and O─Mo─O stretching vibrations in the {Mo_72_Fe_30_} POM nanostructures.^[^ ^40^ ^]^ The other bands observed at 280, 340, 970, and 990 cm^−1^ are raised from the vibrations of Fe─O, Mo─O, Mo(O), and Mo═O_(1)_, respectively.^[^ ^40^, ^41^, ^42^ ^]^ The chemical state of elements at the electrocatalyst surface is important in understanding their electrocatalytic activities. Figure S1 (Supporting Information) shows the typical X‐ray photoelectron survey spectrum of the {Mo_72_Fe_30_} POM nanostructure, which indicated the presence of Mo, Fe, C and O components at ≈232, ≈710, ≈285, and ≈530 eV, respectively. To gain deeper insights from the XPS analysis, the core‐level spectra of individual elements are deconvoluted and presented in Figure 1C–F. The core‐level spectrum of Mo 3d states (Figure 1C) suggested the presence of two peaks at binding energies of 232.5 and 235.7 eV that arise from the Mo 3d_5/2_ and Mo 3d_3/2_ states, respectively.^[^ ^43^, ^44^ ^]^ Figure 1D shows the core‐level spectrum of Fe 2p states, indicating the presence of two major peaks at binding energies of 711.8 and 725.9 eV corresponding to the Fe 2p_3/2_ and Fe 2p_1/2_ states.^[^ ^42^ ^]^ It also possesses satellite peaks at 719.8 and 733.8 eV that rise due to the Fe(III) state of the element present in {Mo_72_Fe_30_} POM, similar to Fe_2_O_3_ and FeS_2_.^[^ ^42^, ^45^ ^]^ Additionally, the presence of peaks at 713.2, 715.96, and 728.6 eV indicated the presence of Fe^III^ valence states.^[^ ^46^ ^]^ The core‐level spectrum of C 1s and O 1s states of elements present in {Mo_72_Fe_30_} POM is located at 284.5 and 532 eV, as seen in Figure 1E,F.

**Figure 2:**
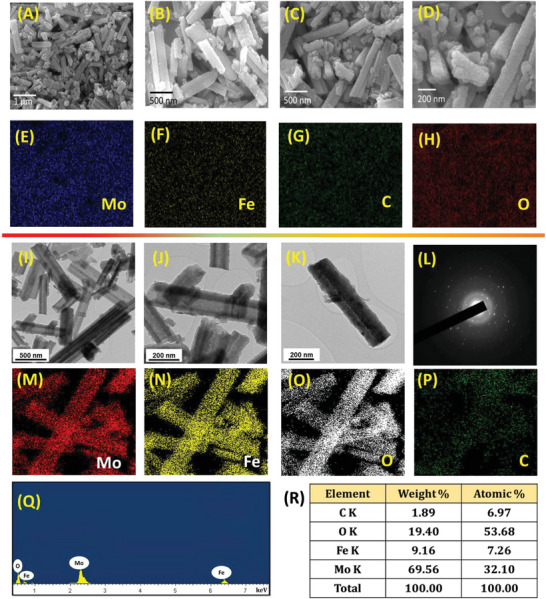
Morphological and elemental analysis of {Mo_72_Fe_30_} POM nanostructures. A–D) Field‐emission scanning electron micrographs (FE‐SEM) obtained under different magnifications, E–H) Elemental maps of E) molybdenum (Mo), F) iron (Fe), G) carbon (C), and H) oxygen (O), I–K) High‐resolution transmission electron micrographs (HR‐TEM) recorded under different magnification level, L) Selected area diffraction pattern, M–P) Elemental maps of M) Mo, N) Fe, O) O, and P) C elements, Q) Elemental spectrum, and R) composition analysis, respectively.

Surface morphology and composition of the electrocatalyst play a vital role in their electrocatalytic performance; therefore, in this work, we have characterized the {Mo_72_Fe_30_} POM using FE‐SEM and HR‐TEM equipped with EDX analysis, respectively. **Figure** **2**A–D depicts the presence of rod‐like nanostructures composed of the {Mo_72_Fe_30_} POM with irregular length and breadth dimensions, which is evidenced from the high‐magnification micrograph given in Figure 2D. The elemental maps of Mo, Fe, C, and O elements present in the {Mo_72_Fe_30_} POM obtained from the overlay map (Figure S2, Supporting Information) are presented in Figure 2E–H that confirmed the homogeneous distribution of all the elements throughout the nanostructures. The corresponding EDX spectrum of the {Mo_72_Fe_30_} POM nanostructures is provided in Figure S3 (Supporting Information). The HR‐TEM micrograph of {Mo_72_Fe_30_} POM given in Figure 2I–K indicated the formation of nanorods with heterogenous dimensionality. It is evident from Figure 2K that the length and breadth of an individual {Mo_72_Fe_30_} POM nanorod are in the range of ≈800 and ≈150 nm, respectively. Here, it is worth mentioning that the ultrasound irradiation process used in the preparation of {Mo_72_Fe_30_} POM plays a vital role in the formation of nano rod‐like structures because of the acoustic cavitation effect in the synthesis media.^[^ ^47^ ^]^ An earlier study indicated the formation of {Mo_72_Fe_30_} POM nanoclusters using a similar precursor without ultrasound irradiation effect.^[^ ^31^ ^]^ The formation of rod‐like structures with the presence of ultrasound in the reaction medium is in close agreement with our previous studies on metal molybdates,^[^ ^48^ ^]^ whereas {Mo_72_Fe_30_} POM is a subset of molybdate‐based oxides containing Fe atoms at their interstitial sites bonded with oxygen. Figure 2L presents the SAED pattern of the {Mo_72_Fe_30_} POM, indicating the presence of a ring‐like pattern because of their polycrystalline nature. Figure 2M–P summarizes the uniform distribution of Mo, Fe, C, and O elements in the {Mo_72_Fe_30_} POM using HR‐TEM maps, and the corresponding spectrum is provided in Figure 2Q. Based on the Cliff–Lorimer thin ratio section analysis method, the elemental composition of Mo:Fe:C:O is in the range of 69.56:9.16:1.89:19.4, as seen in Figure 2R. Figure S4 (Supporting Information) depicts BET results of the {Mo_72_Fe_30_} POM nanostructures from which their surface area and pore size distribution are determined as 11.49 m^2^ g^−1^ and 18.38 nm, respectively.

### 2.2 Electrocatalytic Properties of {Mo_72_Fe_30_} POM Toward HER and OER

**Figure 3:**
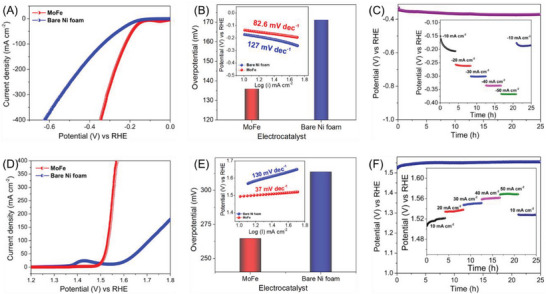
Electrocatalytic performance of {Mo_72_Fe_30_} POM nanostructures toward HER and OER. A) HER polarization curves, B) overpotential analysis and Tafel plots (inset) for HER, C) HER long‐term stability tests over 25 h, and the inset shows the multi‐current analysis over 25 h, D) OER polarization curves, E) overpotential analysis and the Tafel plots (inset) for OER, F) OER long‐term stability tests over 25 h, and the inset shows the multi‐current analysis over 25 h.

The electrocatalytic activity of {Mo_72_Fe_30_} POM electrocatalyst toward HER and OER was studied at the electrode level using a three‐electrode configuration via standard tests such as polarization curves, multi‐current analysis, and long‐term stability.^[^ ^3^ ^]^ **Figure** **3**A shows the HER polarization curves of {Mo_72_Fe_30_} POM electrocatalyst compared to bare Ni foam, which indicates that the former possesses better catalytic activity than the latter. It is recommended to analyze the catalytic performance of an electrocatalyst using the required potential (overpotential) to drive a current density of 10 mA cm^−2^, respectively, based on the standard benchmark evaluation methods.^[^ ^3^ ^]^ Figure 3B shows that the {Mo_72_Fe_30_} POM electrocatalyst possesses a lower overpotential of ≈135 mV to drive a current density of 10 mA cm^−2^, which is lower compared to that of bare Ni foam (171.19 mV). The inset of Figure 3B evidences that the {Mo_72_Fe_30_} POM electrocatalyst possesses a small Tafel slope of 82.6 mV dec^−1^, which was lower than the bare Ni foam (127 mV dec^−1^), respectively. Based on the classical theory and the observed Tafel slope, it can be proposed that the observed HER occurred at the {Mo_72_Fe_30_} POM electrocatalyst follows the Volmer–Heyrovsky mechanism as explained using the following relations:^[^ ^49^ ^]^

(1) $$\begin{array}{c} \left\{Mo_{72}Fe_{30}\right\}\;\mathrm{POM}+H_{2}O+e^{-}\to \left\{Mo_{72}Fe_{30}\right\}\;\mathrm{POM}\;H_{\mathrm{ads}}+OH^{-} \end{array}$$

(2) $$\begin{array}{ccc} \left\{Mo_{72}Fe_{30}\right\}\;\mathrm{POM}\;H_{\mathrm{ads}} & & +H_{2}O+e^{-}\to \left\{Mo_{72}Fe_{30}\right\}\;\\ & & \times \mathrm{POM}+H_{2}+OH^{-} \end{array}$$

Long‐term stability and multi‐current analysis are important parameters determining an electrode's electrochemical stability and practical applicability for effective HER.^[^ ^50^ ^]^ Figure 3C depicts the electrochemical stability of the {Mo_72_Fe_30_} POM electrocatalyst via monitoring the output potential ranges over 25 h by applying a current density of −50 mA cm^−2^. It showed that potential ranges changed from −331 to −370 mV (vs RHE) after 25 h of uninterrupted measurements, thus highlighting their better electrochemical stability.^[^ ^51^ ^]^ The HER multi‐current analysis of the {Mo_72_Fe_30_} POM electrocatalyst over 25 h measured using different steps of current densities (from −10 to −50 mA cm^−2^) is given in the inset of Figure 3C. The current ramping from −10 to −50 mA cm^−2^ regains the initial potential ranges even after 25 h of uninterrupted measurements, thus highlighting their rate capability for various load levels. These studies highlight the better HER activity of the prepared {Mo_72_Fe_30_} POM electrocatalyst compared to the state‐of‐the‐art electrocatalyst materials reported in the literature, as given in Table S1 (Supporting Information). To explore the bi‐functional catalytic activity of the {Mo_72_Fe_30_} POM electrocatalyst, we have examined their properties toward OER, and the results are summarized in Figure 3D–F. The OER polarization curves provided in Figure 3D suggested the better OER properties of {Mo_72_Fe_30_} POM electrocatalyst compared to that of the bare Ni foam. The {Mo_72_Fe_30_} POM electrocatalyst possesses a low overpotential of ≈264 mV compared to that of bare Ni foam (313 mV), respectively, as seen in Figure 3E. The obtained overpotential value of {Mo_72_Fe_30_} POM electrode is better compared to many of the recently reported electrocatalysts for OER, as seen in Table S2 (Supporting Information). Interestingly, the Tafel slope of {Mo_72_Fe_30_} POM electrocatalyst determined from the polarization curves is 37 mV dec^−1^ (as seen from the inset of Figure 3E), which is far better compared to that of Ni foam (130 mV dec^−1^). Figure 3F depicts the electrochemical stability of 25 h of {Mo_72_Fe_30_} POM electrocatalyst, which showed excellent stability with no significant changes observed in the potential values. Similarly, the multi‐current analysis of {Mo_72_Fe_30_} POM electrocatalyst (inset of Figure 3F) shows that the potential values were retained even after repeated cycling with various current ranges. Figure S5A (Supporting Information) depicts the Nyquist plot of the {Mo_72_Fe_30_} POM electrocatalyst measured under OCP conditions, revealing the presence of a quasi semi‐circle at a high frequency (due to charge transfer resistance) followed by a straight line (Warburg impedance) at the low‐frequency regime. The solution resistance (*R_s_*) of the {Mo_72_Fe_30_} POM electrocatalyst is found to be 0.73 Ω, which is used for the IR compensation in the LSV analysis. The Nyquist plot of the {Mo_72_Fe_30_} POM electrocatalyst measured under fixed potentials of ‐0.25 V and +1. 5 V vs RHE (Figure S5B, C, Supporting Information) shows that there are no significant changes occurred in the *R_s_* value in comparison to that of Figure S5A (Supporting Information). However, the Warburg impedance is diminished, and a clear semi‐circle region with *R_ct_* values of 1.49 and 0.29 Ω, were obtained for the {Mo_72_Fe_30_} POM electrocatalyst measured under an applied potential of ‐0.25 V vs RHE and +1.5 V vs RHE, respectively. The obtained *R_ct_* values of the {Mo_72_Fe_30_} POM electrocatalysts were lower compared to the reported works in literature ^[^ ^52^, ^53^, ^54^ ^]^, thus facilitating the HER and OER reactions. Additionally, the electrochemical active surface area (ECSA) of the {Mo_72_Fe_30_} POM electrocatalyst are also examined in this work. The ECSA of the {Mo_72_Fe_30_} POM electrocatalyst was determined from the electric double‐layer capacitance measurements in 1 M KOH electrolyte.

The cyclic voltammograms (CV) of {Mo_72_Fe_30_} POM electrocatalyst recorded at different scan rates are provided in Figure S6A (Supporting Information), which showed the presence of rectangular‐shaped profiles as an indication of double‐layer capacitance. Further, the current ranges (both anodic and cathodic) are increased with an increase in scan rate from 10 to 150 mV s^−1^, as evident from Figure S6B (Supporting Information). From this, the double layer capacitance (*C*_dl_) and ECSA of the {Mo_72_Fe_30_} POM electrode were determined as 1.96 µF and 49 cm^2^, respectively.

### 2.3 Understanding the Bi‐Functional Catalytic Properties of {Mo_72_Fe_30_} POM Using SECM and Electrochemical Gating Methods

**Figure 4:**
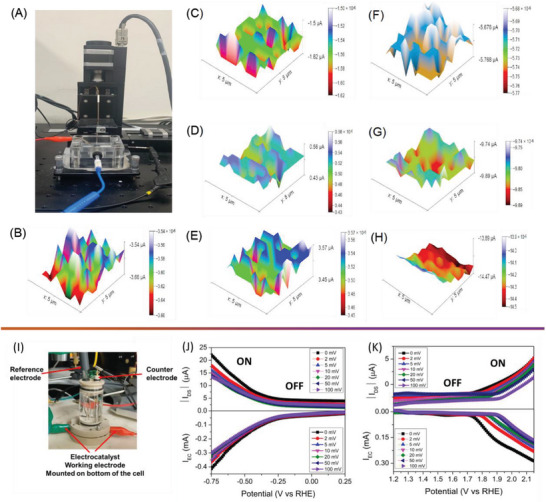
A–H) Scanning electrochemical microscope (SECM) analysis of {Mo_72_Fe_30_} POM electrocatalyst toward HER and OER. A) Digital photograph of Sensolytics SECM, (B‐H) Tip current map recorded at different applied bias potentials viz., B) 0 mV, C) −500 mV, D)−1000 mV, E) −1500 mV, F) +500 mV, G)+1000 mV, and H) +1500 mV, respectively. The potential values are provided against Ag/AgCl reference electrode. I) Digital photograph of the electrolyte‐gated transistor‐bottom magnetic mount cell (obtained from Redox.me) used for the measurement of electrochemical gating in {Mo_72_Fe_30_} POM electrocatalyst. J,K) Electrochemical gating analysis of {Mo_72_Fe_30_} POM electrocatalyst toward J) HER and K) OER in which the electrochemical (*I*_EC_) and electrical (*I*_DS_) signals are monitored under different bias potentials from 2 to 50 mV.

To further explore the intrinsic electrocatalytic properties of {Mo_72_Fe_30_} POM, we have used SECM and electrochemical gating methods in this work. It is worth mentioning that SECM is one of the important electroanalytical techniques used for imaging the topography of nanostructured electrode materials and their local reactivity in a high resolution.^[^ ^55^, ^56^ ^]^ **Figure** **4**A portrays the digital photograph of the SECM that is operated using the substrate generation and tip collection (SG‐TC) mode using a bi‐potentiostat. Herein, the substrate and the tip currents were measured simultaneously, in which the former is used to monitor the cell current ({Mo_72_Fe_30_} POM electrocatalyst), whereas the latter is used to monitor the generated H_2_ and O_2_ gases, respectively. Figure S7 (Supporting Information) shows the substrate current generated from the {Mo_72_Fe_30_} POM electrocatalyst under 0 V applied bias potential, and the corresponding tip current map is provided in Figure 4B. Under a biasing voltage of 0 V (Figure 4B), the surface currents generated from the {Mo_72_Fe_30_} POM electrocatalyst and the tip currents are found to be 0.08 mA and −3.58 µA; these values are low since there is no significant electrochemical catalytic reactions occurred at their surface. The substrate current maps of the {Mo_72_Fe_30_} POM electrocatalyst recorded at different applied bias potentials are provided in Figure S8 (Supporting Information). The substrate current of the {Mo_72_Fe_30_} POM electrocatalyst is found to be 0.08 mA under applied bias potential of 0.0 V (Figure S7, Supporting Information), which was linearly increased with a corresponding increase in bias potential from 0.0 V into ±500, ±1000, and ±1500 mV, respectively. The observed maximal HER current of the {Mo_72_Fe_30_} POM electrocatalyst from the substrate current maps are found to be −0.03, −1.05, and −143.8 mA under biasing at −500, −1000, and −1500 mV, respectively. Figure 4C–E presents the tip current generated as a result of the hydrogen oxidation reaction at the surface of the platinum ultra‐microelectrode (UME). At different bias potentials of the {Mo_72_Fe_30_} POM electrocatalyst, such as 0, −500, −1000, and −1500 mV, the tip currents are found to be −3.60, −1.55, +0.52, and 3.5 µA, respectively. The increase in the tip current from low to high applied potentials is due to the oxidation of more H_2_ gases at high bias potentials. From Figure S9 (Supporting Information), the observed maximal OER current of the {Mo_72_Fe_30_} POM electrocatalyst from the substrate current maps are found to be +10.64, +322, and +681 mA under biasing at +500, +1000, and +1500 mV, respectively. From the 3D array current maps given in Figure 4F,G, the generated tip currents as a result of oxygen reduction reaction are in the range from −5.67 to −5.76 µA (+500 mV vs Ag/AgCl) and from −9.74 to −9.89 µA (+1000 mV vs Ag/AgCl) only. Further, at higher biasing voltage, high currents (because of OER) were seen from the 3D maps given in Figure 4H, such as from −13.49 to −14.87 µA (+1500 mV vs Ag/AgCl) respectively.

Next, we studied the effect of the electrochemical self‐gating phenomenon to understand the electrochemical catalytic properties of the {Mo_72_Fe_30_} POM nanostructures–electrolyte interfaces toward effective HER and OER. Previously, many theories and models, such as the Schottky‐analogue junction model (for interface studies) and Marcus theory/Gerischer model (for electron transfer), were used for semiconductor electrocatalysis, whereas these models are not suitable for ultrathin sub‐nanometric size semiconductors.^[^ ^39^, ^57^ ^]^ Later, Y. He et al. demonstrated a new model based on the self‐gating phenomenon as promising for understanding semiconductor electrocatalysis.^[^ ^39^ ^]^ Recently, few works demonstrated the use of electrochemical gating for understanding the electrochemical catalytic (either HER or OER) properties of various 2D sheets (monolayer MoS_2_, 1T’‐WTe_2_, 2D metal phosphides), respectively.^[^ ^58^, ^59^, ^60^, ^61^ ^]^ In this work, we have utilized the electrochemical gating effect to understand the electrochemical reactions that occurred at the interface between {Mo_72_Fe_30_} POM electrode‐electrolyte. The circuit diagram used for the four‐terminal measurement and the electrochemical gating cell are provided in Figure S10 (Supporting Information). Here, simultaneous measurement of electrochemical current (*I*_EC_) in the 3E cell and the electrical signals (*I*_DS_) from the electrocatalyst materials during the HER and OER process was monitored to understand the mechanism of bipolar activities via the role of different charge carriers (electrons and holes), respectively. The experimental setup used for studying the electrochemical gating effect at {Mo_72_Fe_30_} POM electrode‐electrolyte interface is shown in Figure 4I. Here, the {Mo_72_Fe_30_} POM electrocatalyst is placed at the bottom of the cell, whereas the reference and counter electrodes are mounted at the top of the cell. Figure 4J shows the variation of *I*_EC_ and *I*_DS_ against the voltage (V vs RHE) recorded at different electrode potentials (from 0 to 100 mV). It showed that the *I*_EC_ (due to the HER) process varies significantly with respect to the applied electrochemical potential (from 0.0 V. to −0.750 V vs RHE) with the onset potential at the range of −0.26 V (V vs RHE). It is known that under negative electrochemical potential, the electrons will transfer from the current collector to the outermost surface of the electrocatalyst to drive the HER.^[^ ^62^, ^63^ ^]^ Thus, the simultaneous electric signals from the {Mo_72_Fe_30_} POM electrocatalyst (Figure 4J, top panel) show the presence of conductance modulation at the region higher than the HER onset potential results in turn “ON” condition, that is, a more conductive state favoring the cathodic HER process. This clearly indicated that the overall electrochemical reactions occurred only when the {Mo_72_Fe_30_} POM electrocatalyst was turned on by the electrochemistry‐driven self‐gating process.^[^ ^39^ ^]^ It showed the threshold voltage of −32 mV (vs RHE) obtained for the {Mo_72_Fe_30_} POM electrocatalyst with an ON/OFF ratio of 0.15 × 10^2^, respectively. Similarly, the variation of *I*_EC_ and *I*_DS_ against the voltage (V vs RHE) in the OER region is presented in Figure 4K, indicating the conductance modulation of the majority charge carrier (holes) during the OER process. The threshold voltage of {Mo_72_Fe_30_} POM electrocatalyst during the OER process is determined to be 1740 mV (vs RHE) with an ON/OFF ratio of 0.18 × 10^2^, respectively. Previous studies indicated that unipolar (either n‐type or p‐type) materials dominate either the HER or OER processes, whereas bipolar materials can drive both of these reactions.^[^ ^39^ ^]^ The presence of two threshold voltages in the {Mo_72_Fe_30_} POM electrocatalyst further confirmed their bipolar nature; thereby, bi‐functional catalytic properties originated. These observations were in good agreement with the previous works on bi‐functional catalytic properties of the WSe_2_ sheets.^[^ ^39^ ^]^

### 2.4 Performance Assessment of {Mo_72_Fe_30_} POM‐Based Water Electrolyzer and Self‐Powered System

**Figure 5:**
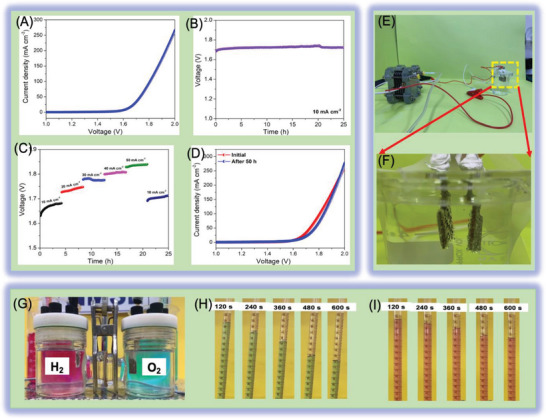
Performance evaluation of prototype water electrolyzer made of {Mo_72_Fe_30_} POM nanostructures as bi‐functional electrocatalyst. A) Two‐electrode polarization curves, B) Stability analysis, C) Multi‐current analysis recorded at different current densities, D) Polarization curves recorded after 50 h of electrochemical water splitting, E) Demonstration of self‐powered system comprising DMFC driven {Mo_72_Fe_30_} POM water electrolyzer, and F) shows magnified portion indicating the production of hydrogen and oxygen bubbles at the surface of {Mo_72_Fe_30_} POM electrocatalyst, G–I) Real‐time H‐type hybrid membrane water electrolyzer setup was used to collect the hydrogen gas (H_2_) and oxygen gas (O_2_) from {Mo_72_Fe_30_} POM electrocatalyst with the drainage setup used to evaluate the faradaic efficiency.

The above results highlighted the bi‐functional properties of the {Mo_72_Fe_30_} POM electrocatalyst using 3E tests; however, it is important to study the device‐specific properties of these materials for realizing their practical applications.^[^ ^64^ ^]^ Therefore, we have fabricated a lab‐scale water electrolyzer using two ideal {Mo_72_Fe_30_} POM electrocatalyst for HER and OER, and the corresponding LSV profiles recorded using a 5 mV s^−1^ scan rate are given in **Figure** **5**A. It showed that the {Mo_72_Fe_30_} POM electrolyzer requires only a low voltage of 1.62 V to reach 10 mA cm^−2^ current density with better production of hydrogen and oxygen gases as the surfaces of {Mo_72_Fe_30_} POM electrocatalyst. The cell voltage of the {Mo_72_Fe_30_} POM electrolyzer is remarkably better than the reported electrolyzer based on bi‐functional electrocatalysts in alkaline media (as listed in Table S3, Supporting Information). The electrochemical stability studies of the {Mo_72_Fe_30_} POM electrolyzer (Figure 5B) show that the device requires a voltage of 1.68 V to drive a current density of 10 mA cm^−2^ initially; these values increase up to 1.72 V after 25 h of stability tests. The observed increase in voltage after electrochemical stability tests can be explained because of the partial wettability of the {Mo_72_Fe_30_} POM electrocatalyst surfaces that led to the formation of a new electrolyte/electrode interfacial environment.^[^ ^54^ ^]^ Figure 5C depicts the multi‐current analysis of the {Mo_72_Fe_30_} POM electrolyzer, which indicated that they are capable of driving water electrolysis reactions at different load ranges. Figure 5D presents the LSV analysis of the {Mo_72_Fe_30_} POM electrolyzer after 50 h of uninterrupted electrochemical reactions recorded at 5 mV s^−1^ scan rate, respectively. It indicated that there is no noticeable change in the overpotential values because of better electrochemical stability of the bi‐functional {Mo_72_Fe_30_} POM electrocatalysts present in the fabricated water electrolyzer. Figures S11 and S12 (Supporting Information), and its associated discussion presents the morphological analyses of the {Mo_72_Fe_30_} POM electrocatalysts before and after electrochemical stability tests, which further ensures their structural robustness even after long‐term stability tests. Figure 5E demonstrates the self‐powered system via the integration of a 2.0 V DMFC driven {Mo_72_Fe_30_} POM water electrolyzer. The open circuit voltage and polarization curves of the DMFC (given in Figure S13, Supporting Information) show that they can provide a constant voltage of 2.2 V with a peak power of 40 mW, respectively. The DMFC‐powered {Mo_72_Fe_30_} POM water electrolyzer indicated the uninterrupted production of hydrogen and oxygen bubbles (Figure 5F) at the surface of {Mo_72_Fe_30_} POM electrocatalyst. Figure 5G–I and Video S1 (Supporting Information) presents the experimental setup (water drainage method) used for the measurement of the produced H_2_ and O_2_ gases from the bi‐functional {Mo_72_Fe_30_} POM water electrolyzer in H‐cell type configuration driven by a constant current density of 100 mA cm^−2^.

### 2.5 Realization of {Mo_72_Fe_30_} POM‐Based Water Electrolyzer Under Industrial Conditions

**Figure 6:**
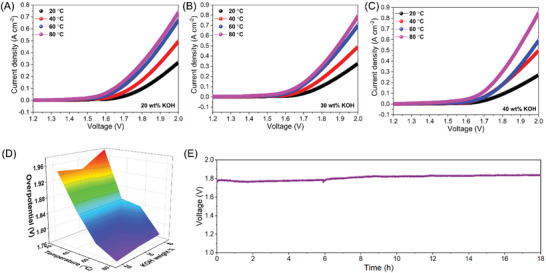
Electrochemical analysis of {Mo_72_Fe_30_} POM‐based industrial water electrolyzer. A–C) LSV profiles recorded under different concentrations of KOH electrolyte (20%, 30%, and 40%) with respect to temperature (20, 40,60, and 80 °C), D) Plot of overpotential value required to drive a current of 250 mA cm^−2^ with respect to different temperature and KOH concentrations, and E) stability analysis over 18 h, respectively.

It is worth mentioning that the reliability of the current benchmarks used in lab‐scale electrocatalysis research, such as overpotential (@10 mA cm^−2^, metric for solar fuel conversion),^[^ ^65^ ^]^ is quite questionable or challenging the industrial requirements.^[^ ^66^ ^]^ Further, the testing conditions also have significant differences from the lab‐scale research to industrial requirements, in which the latter requires performance evaluation under elevated temperatures (40–80 °C) and higher concentrations of KOH (20%–40%).^[^ ^66^, ^67^ ^]^ Figures S14 and S15 (Supporting Information), SI shows the HER and OER profiles of {Mo_72_Fe_30_} POM electrocatalyst based on the conditions suitable for industrial alkaline electrolyzer. The HER overpotential required to drive 500 mA cm^−2^ is ≈607, 539, 502, and 492 mV (vs RHE) for the {Mo_72_Fe_30_} POM electrocatalyst at a temperature of 20, 40, 60, and 80 °C with 40% KOH. Similarly, the OER overpotential required to drive 500 mA cm^−2^ is ≈330, 252, 193, and 172 mV (vs RHE) for the {Mo_72_Fe_30_} POM electrocatalyst at a temperature of 20, 40, 60, and 80 °C with 40% KOH. The increase in current ranges in the HER and OER polarization curves at industrial standard conditions in comparison to the laboratory standard conditions might be due to the higher electrolyte ion conductivity of the KOH (20%–40%) at elevated temperatures compared to 1 m KOH typically used in lab‐scale research.^[^ ^66^, ^68^ ^]^ **Figure** **6**A–C presents the LSV profiles of {Mo_72_Fe_30_} POM water electrolyzer measured at the required conditions of an industrial alkaline electrolyzer. It revealed that an increase in temperature leads to much higher current ranges with respect to the applied bias voltage and reduction in the device's overpotential. The effect of temperature and KOH concentration on the overpotential of the {Mo_72_Fe_30_} POM water electrolyzer to drive 250 mA cm^−2^ is presented in Figure 6D. The {Mo_72_Fe_30_} POM water electrolyzer requires an overpotential of ≈1.96 and 1.89 V to drive 500 mA cm^−2^ at a temperature of 60 and 80 °C using 40% KOH. The electrochemical stability studies of the {Mo_72_Fe_30_} POM electrolyzer using 40% KOH at 60 °C (Figure 6E) show that the device requires a voltage of 1.76 V to drive a current density of 100 mA cm^−2^ initially; these values increase up to 1.83 V after 18 h of stability test. Strikingly, the overpotential required for the {Mo_72_Fe_30_} POM water electrolyzer, with its good stability, ensures that it might be a promising candidate for the development of next‐generation alkaline electrolyzer systems.

## 3 Conclusion 

The key findings of this work demonstrated the use of {Mo_72_Fe_30_} POM nanostructures as a non‐precious metal‐free bi‐functional electrocatalyst for electrochemical HER and OER with low overpotentials and Tafel slope. The experimental results of the scanning electrochemical microscope showed a direct visualization of catalytic currents (with almost similar magnitude) generated at the surface of nano‐electrocatalysts during the cathodic and anodic reactions, respectively. The bipolar nature of {Mo_72_Fe_30_} POM electrocatalyst was further confirmed using electrochemical gating technology by bridging the electrochemical and electrical signals raised from the 3E cell and electrode. The device‐specific properties of {Mo_72_Fe_30_} POM‐based water electrolyzer showed that they could drive 10 mA cm^−2^ with a much lower voltage of 1.62 V, and a self‐powered system comprising DMFC powered {Mo_72_Fe_30_} POM water electrolyzer is also demonstrated. The performance assessment of {Mo_72_Fe_30_} POM‐based industrial water electrolyzers showed that they require a very low overpotential of 1.89 V to drive 500 mA cm^−2^ (better compared to the state‐of‐the‐art devices) that further ensures their candidature toward the production of clean hydrogen fuel.

## 4 Experimental section

### Materials

The chemicals such as sodium molybdate, ferric chloride, and potassium hydroxide (KOH) were purchased from Daejung Chemicals and Metals Co. Ltd., South Korea. The chemicals utilized in this research work were research‐grade, and only doubly distilled water was used throughout the experiments. The ultrasound irradiation was performed using a probe‐type sonicator (SONICS VCX‐750 model, 20 kHz, 750 W) equipped with a direct immersion titanium horn.

### Preparation of {Mo_72_Fe_30_} POM Nanostructures

The {Mo_72_Fe_30_} POM nanostructures were prepared using a sonochemical approach using a modified method in the literature.^[^ ^31^, ^69^ ^]^ Briefly, the appropriate amount of sodium molybdate was dissolved in deionized water containing glacial acetic acid using ultrasound irradiation, followed by the stepwise addition of a solution containing ferric chloride hexahydrate. The reaction was continued for over 30 min. After that, the resulting powders were washed with DI water via repeated centrifugation and allowed to dry at 80 °C overnight. Finally, the precipitate of {Mo_72_Fe_30_} POM nanostructures was obtained and is used for further studies.

### Instrumentation

X‐ray diffraction pattern of {Mo_72_Fe_30_} POM nanostructures was recorded on Malvern Panalytical instrument, UK, using Cu Kα radiation. The laser Raman spectrum of {Mo_72_Fe_30_} POM nanostructures was acquired using the LabRam‐HR‐Evolution Raman instrument (Horiba Jobin‐Yvon, France) with a laser wavelength of 514 nm. The chemical states of elements present in {Mo_72_Fe_30_} POM nanostructures were studied using X‐ray photoelectron spectroscopy (XPS) analysis (ESCA‐2000 VG Microtech Ltd., KBSI, Busan center). The surface morphology of the {Mo_72_Fe_30_} POM nanostructures was measured using field‐emission scanning electron microscopy (FE‐SEM) equipped with an energy‐dispersive X‐ray (EDX) ‐spectroscopy analyzer (TESCAN, MIRA3) and high‐resolution transmission electron microscopy (HR‐TEM), JEOL JEM 2011, JEOL Ltd., KBSI, Daegu center. The surface area and pore‐size analysis of the {Mo_72_Fe_30_} POM nanostructures were measured on Belsorp Mini X instruments.

### Electrocatalytic Performance Assessment

The {Mo_72_Fe_30_} POM working electrodes were prepared using the slurry coating method. Briefly, {Mo_72_Fe_30_} POM powders were mixed with PVDF binder in the weight ratio 95:5 and grounded with an agate mortar using NMP as a solvent until a homogeneous slurry was formed. The well‐formed slurry was then brush coated on the pre‐cleaned nickel foam with an active area of 1 × 1 cm^2^ and dried at 70 °C overnight. The HER and OER properties of {Mo_72_Fe_30_} POM electrocatalyst (working electrode) were analyzed on an AUTOLAB PGSTAT302N workstation in a three‐electrode (3E) configuration using Ag/AgCl reference electrode and platinum‐foil counter electrodes, respectively. The HER and OER polarization curves were obtained using linear sweep voltammetry (LSV) at a 10 mV s^−1^ scan rate. The potential values were measured against the Ag/AgCl electrode and converted into a reversible hydrogen electrode (RHE) using the relation given in the literature.^[^ ^6^ ^]^ The electrochemical impedance spectroscopy (EIS) analysis was performed in the frequency range 100 mHz–100 kHz at fixed potentials viz. i) open circuit potential (OCP), ii) −0.25 V and iii) +1.5 V Vs. RHE, using an amplitude of 10 mV. The ohmic potential drop (iR) was compensated using the solution resistance (*R*_s_) value measured using electrochemical impedance spectroscopy (EIS) analysis. Electrochemical stability tests were performed by applying a current density of ±50 mA cm^−2^ using chronopotentiometry. Multi‐current analyses of {Mo_72_Fe_30_} POM electrocatalyst were performed using chronopotentiometry by applying various current densities (from ±10 to ±50 mA cm^−2^). The overpotential, and Tafel slope of the {Mo_72_Fe_30_} POM electrocatalyst were calculated using the method given in the earlier work.^[^ ^6^ ^]^

### Scanning Electrochemical Microscopic Analysis of {Mo_72_Fe_30_} POM Electrocatalyst for HER and OER

A Sensolytics interface (Germany, Model No: SECM087) with Autolab‐PGSTAT‐302N workstations was used to carry out the SECM measurements with platinum ultra‐microelectrode (diameter = 10 µm) tip. In a typical cell, the {Mo_72_Fe_30_} POM electrocatalyst were placed at the bottom of the chamber, whereas platinum ring and Ag/AgCl are used as counter and reference electrodes, respectively. All the electrochemical measurements were carried out in 1 m KOH solution. For measuring the SECM array scan (current maps), the SECM tip was aligned vertically above the {Mo_72_Fe_30_} POM electrocatalyst. Before the measurement of the array scan, the SECM tip was moved along the +Z direction by 3 µm from the surface of {Mo_72_Fe_30_} POM electrocatalyst and moved along the X‐Y direction (at a scan rate of 5 µm s^−1^) to avoid any accidental brushing of the tip to the surface of {Mo_72_Fe_30_} POM electrocatalyst. The SECM substrate ({Mo_72_Fe_30_} POM electrocatalyst) was biased at different voltages (±500, 1000, 1500, 2000 mV) versus Ag/AgCl, and the corresponding SECM array current maps of electrode and tip were obtained.

### Electrochemical Gating Analysis of {Mo_72_Fe_30_} POM Electrocatalyst for HER and OER

An electrolyte‐gated transistor‐bottom magnetic mount cell purchased from Redoxme AB, Sweden, was used for the electrochemical gating analysis of {Mo_72_Fe_30_} POM nanostructures toward HER and OER. Here, the {Mo_72_Fe_30_} POM nanostructures were coated on the glass substrates using platinum‐thin films as the current collector was placed at the bottom of the cell. In contrast, the Ag/AgCl reference electrode and platinum ring‐type counter electrodes were placed at the top of the cell. The entire cell was filled with a 1.0 m solution of KOH. To probe the electrochemical signals (*I*_EC_) raised during the HER/OER and the electric signals (*I*_DS_) from the {Mo_72_Fe_30_} POM nanostructures (working electrodes or electrocatalysts), an AUTOLAB PGSTAT302N workstation equipped with a Bi‐potentiostat was used. This instrument facilitates the combination of two independent channels viz., i) electrochemical and ii) electric signals simultaneously. Here, LSV was used to understand the electrochemical signal from the 3E cell using channel‐1. In contrast, channel‐2 monitors the electric signal from the {Mo_72_Fe_30_} POM nanostructures via chronoamperometry at a constant voltage (from 2 to 50 mV), respectively.

### Fabrication, Testing of Lab‐Scale {Mo_72_Fe_30_} POM Water Electrolyzer and Self‐Powered System

A prototype water electrolyzer was fabricated as a beaker‐type cell using the {Mo_72_Fe_30_} POM nanostructure as a bi‐functional electrocatalyst. The LSV profiles, multi‐current analysis, and long‐term stability of the fabricated {Mo_72_Fe_30_} POM‐based water electrolyzer were measured on AUTOLAB PGSTAT302N workstation. To authenticate the practical applicability of the {Mo_72_Fe_30_} POM water electrolyzer, the integration of the water electrolyzer was demonstrated with a commercial 2.0 V DMFC (5‐cell stack purchased from a Fuel cell store, South Korea) as an uninterrupted power source for hydrogen production.

### Performance Assessment of {Mo_72_Fe_30_} POM Water Electrolyzer Under Industrial Conditions

The electrochemical properties of the beaker‐type {Mo_72_Fe_30_} POM water electrolyzer were tested under different concentrations of KOH (20%–40%) and higher temperatures (40, 60, and 80 °C). The polarization curves of the {Mo_72_Fe_30_} POM water electrolyzer were recorded using a scan rate of 5 mV s^−1^, whereas electrochemical stability tests were performed by applying a current density of −100 mA cm^−2^ using chronopotentiometry.

## Conflict of Interest

The authors declare no conflict of interest.

## Supporting information

Supporting Information

Supplemental Video 1

## Data Availability

The data that support the findings of this study are available from the corresponding author upon reasonable request.
