# Supplementary material for: Unravelling the Bi‐Functional Electrocatalytic Properties of {Mo72Fe30} Polyoxometalate Nanostructures for Overall Water Splitting Using Scanning Electrochemical Microscope and Electrochemical Gating Methods
Source: Adv Sci (Weinh). 2024 Apr 12;11(25):2401073. doi: 10.1002/advs.202401073 (PMC11220659; doi:10.1002/advs.202401073)
Supplement: Supplementary file 1 — Supporting Information [file ADVS-11-2401073-s002.pdf]

ADVANCED SCIENCE
Open Access

Supporting Information

for *Adv. Sci.*, DOI 10.1002/advs.202401073

Unravelling the Bi-Functional Electrocatalytic Properties of $\{Mo_{72}Fe_{30}\}$ Polyoxometalate Nanostructures for Overall Water Splitting Using Scanning Electrochemical Microscope and Electrochemical Gating Methods

*Karthikeyan Krishnamoorthy, Parthiban Pazhamalai, Rajavarman Swaminathan, Vigneshwaran Mohan and Sang-Jae Kim**

Supporting Information

Unravelling the bi-functional electrocatalytic properties of {$Mo_{72}Fe_{30}$} polyoxometalate nanostructures for overall water splitting using scanning electrochemical microscope and electrochemical gating methods

*Karthikeyan Krishnamoorthy#, Parthiban Pazhamalai #, Rajavarman Swaminathan, Vigneshwaran Mohan, and Sang -Jae Kim**

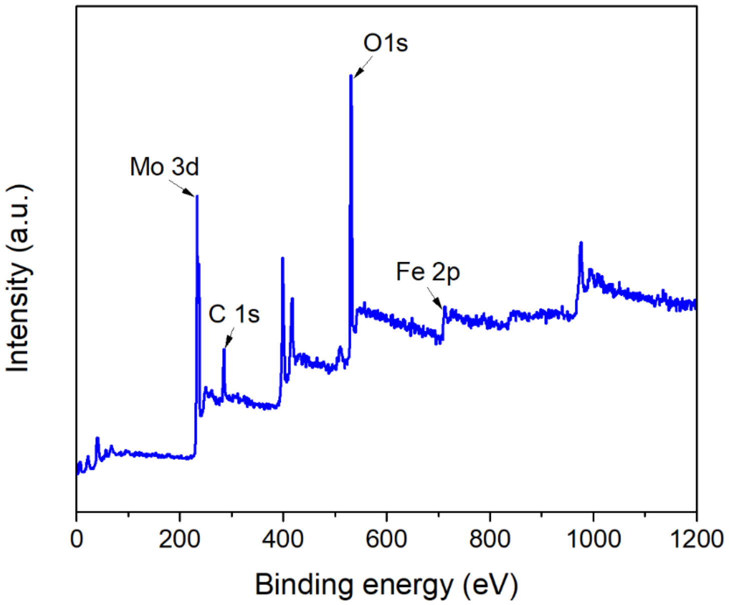

Figure S1. X-ray photoelectron survey spectrum of {$Mo_{72}Fe_{30}$} POM nanostructures.

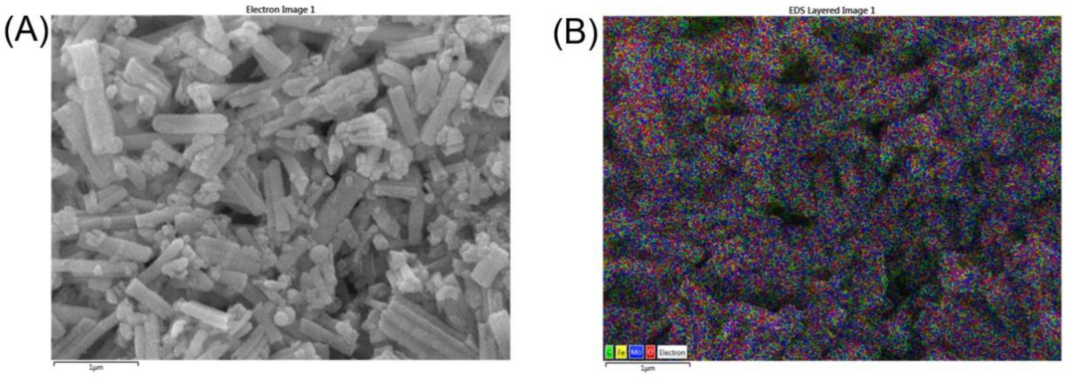

Figure S2. Overlay spectrum used for elemental mapping analysis of $\{Mo_{72}Fe_{30}\}$ POM nanostructures.

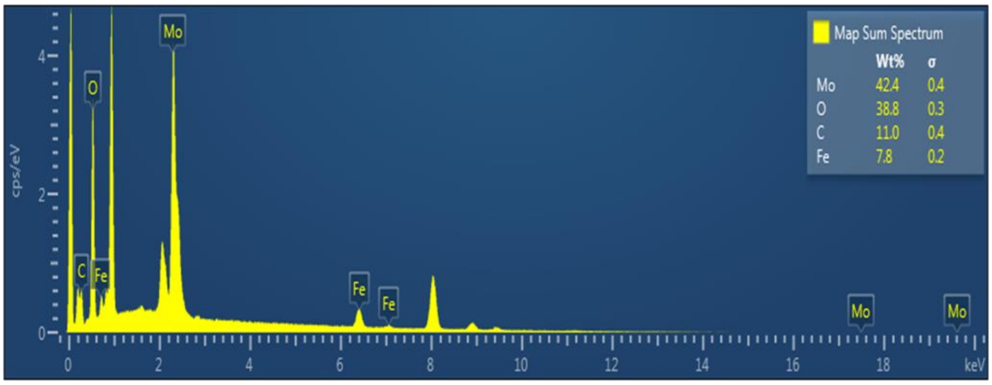

Figure S3. Elemental spectrum of $\{Mo_{72}Fe_{30}\}$ POM nanostructures.

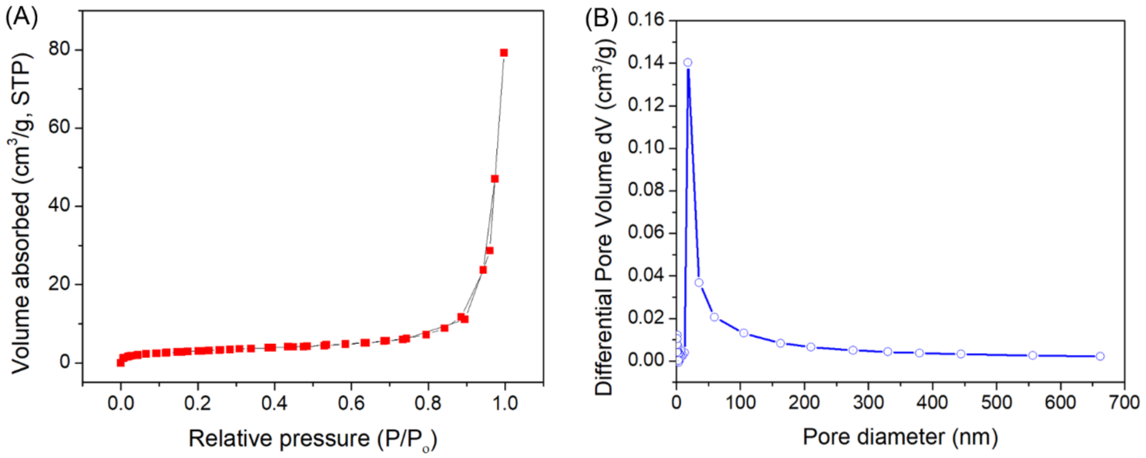

Figure S4. (A) BET surface area and (B) pore size analysis of {$Mo_{72}Fe_{30}$} POM nanostructures.

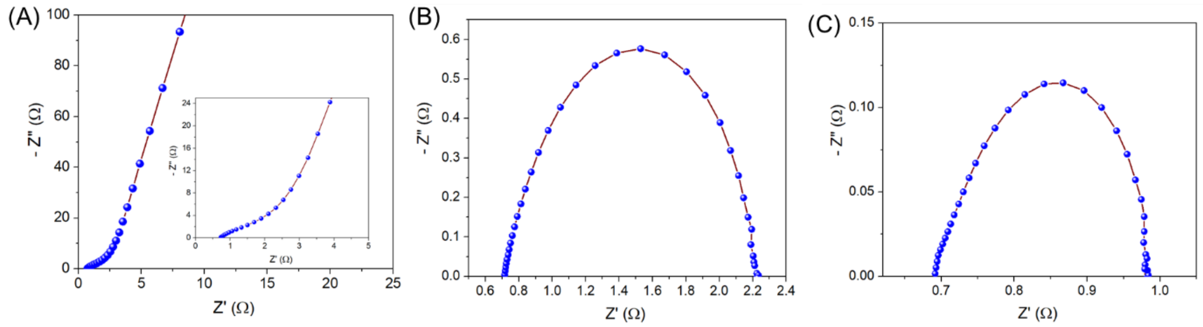

Figure S5. Nyquist plot of the{$Mo_{72}Fe_{30}$} POM electrode measured under (A) OCP condition; (B) -0.25 V vs RHE and (C) +1.5 V vs RHE, respectively.

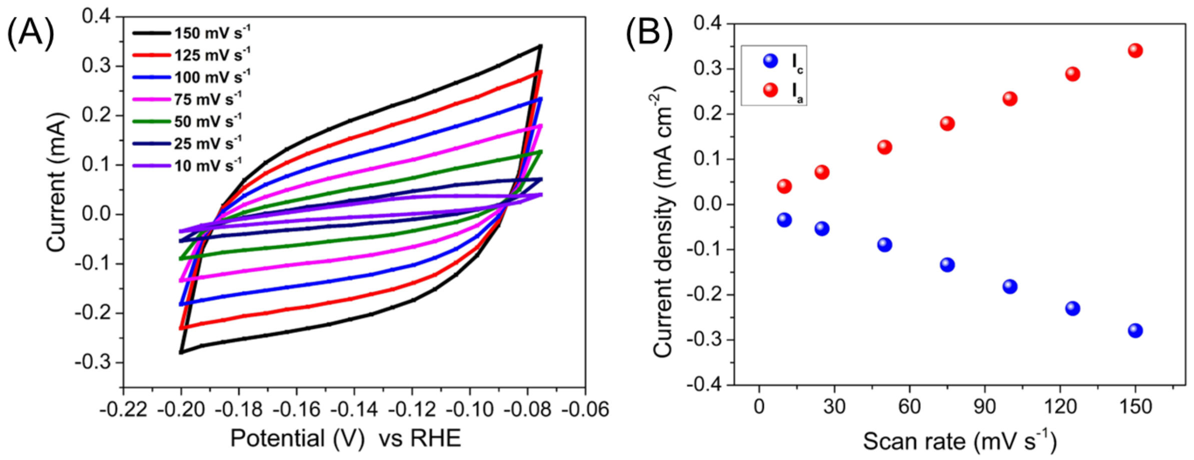

Figure S6. (A) Cyclic voltammetry of {$Mo_{72}Fe_{30}$} POM nanostructures recorded at different scan rates and (b) presents the plot of capacitive currents anodic and cathodic with respect to different scan rates. The electrolyte is 1 M KOH.

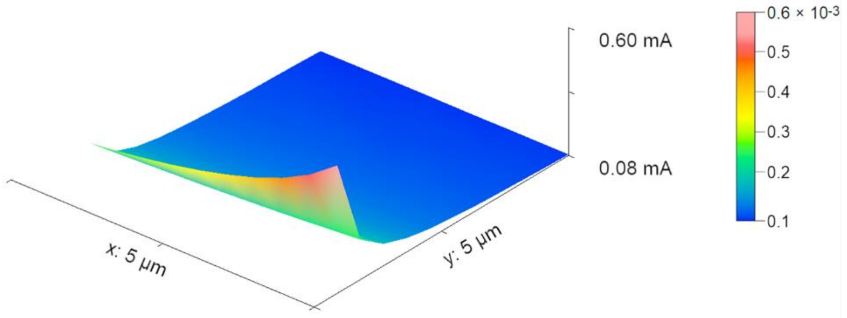

Figure S7. Substrate current generated from the {$Mo_{72}Fe_{30}$} POM nanostructures under 0 V applied bias potential.

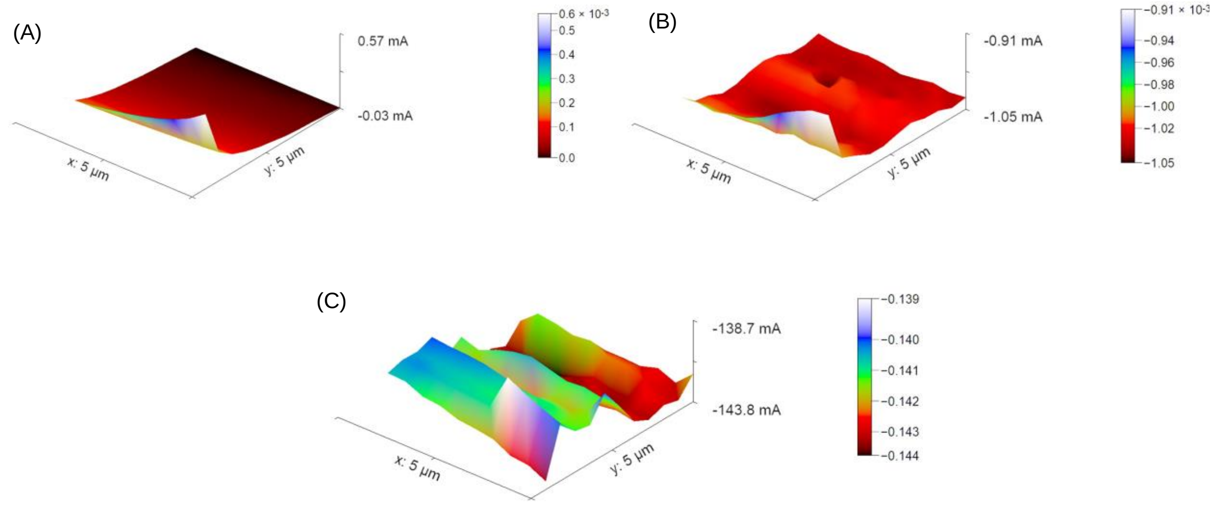

Figure S8. Substrate current generated from the {$Mo_{72}Fe_{30}$} POM nanostructures under different applied bias potentials (-500 mV, -1000 mV, and -1500 mV).

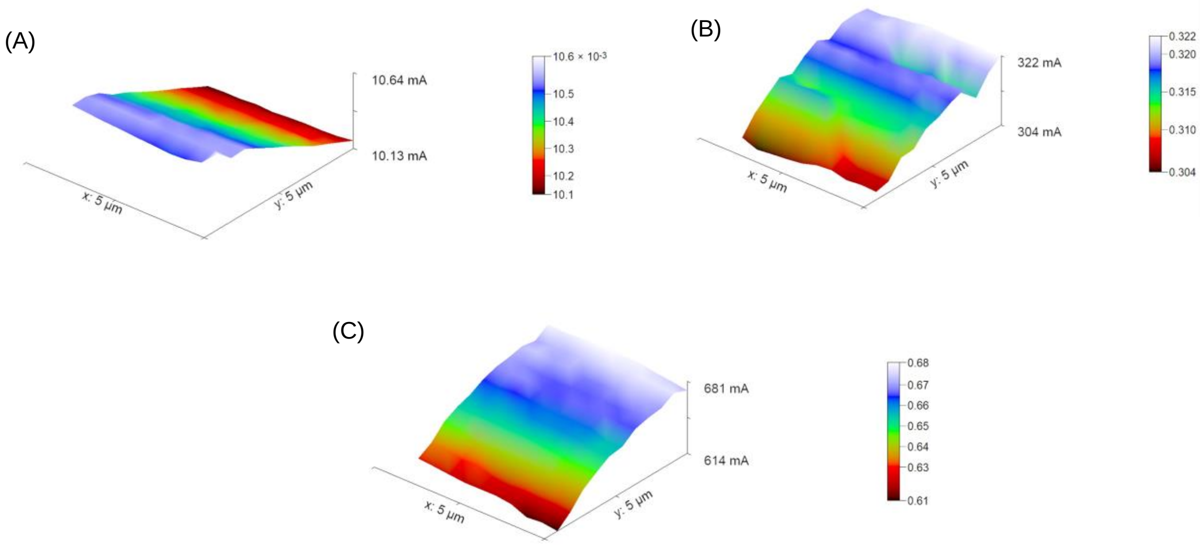

Figure S9. Substrate current generated from the {$Mo_{72}Fe_{30}$} POM nanostructures under different applied bias potentials (+500 mV, +1000 mV, and +1500 mV).

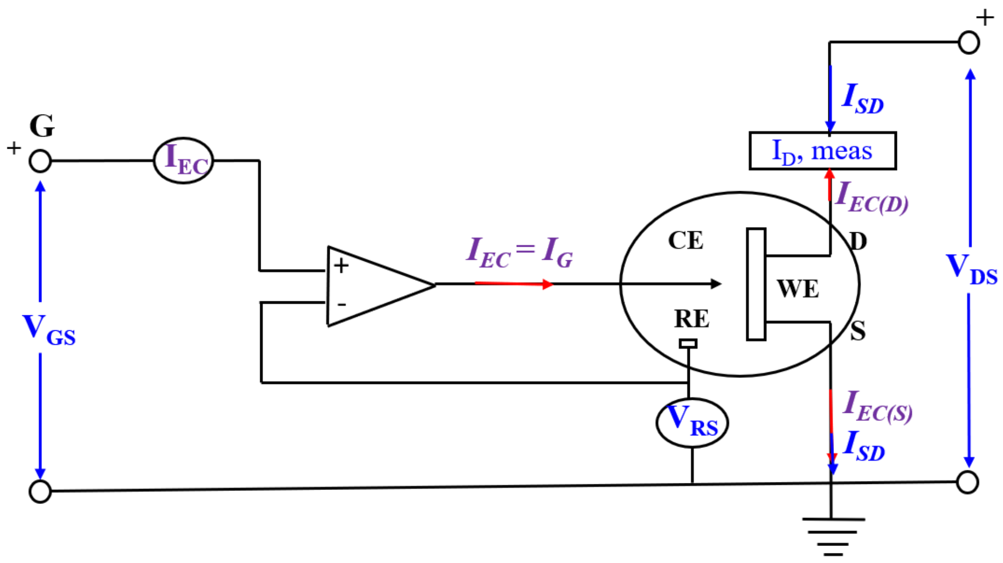

Figure S10. An equivalent circuit used for the electrochemical gating analysis of bifunctional catalytic property of the {$Mo_{72}Fe_{30}$} POM nanostructures.

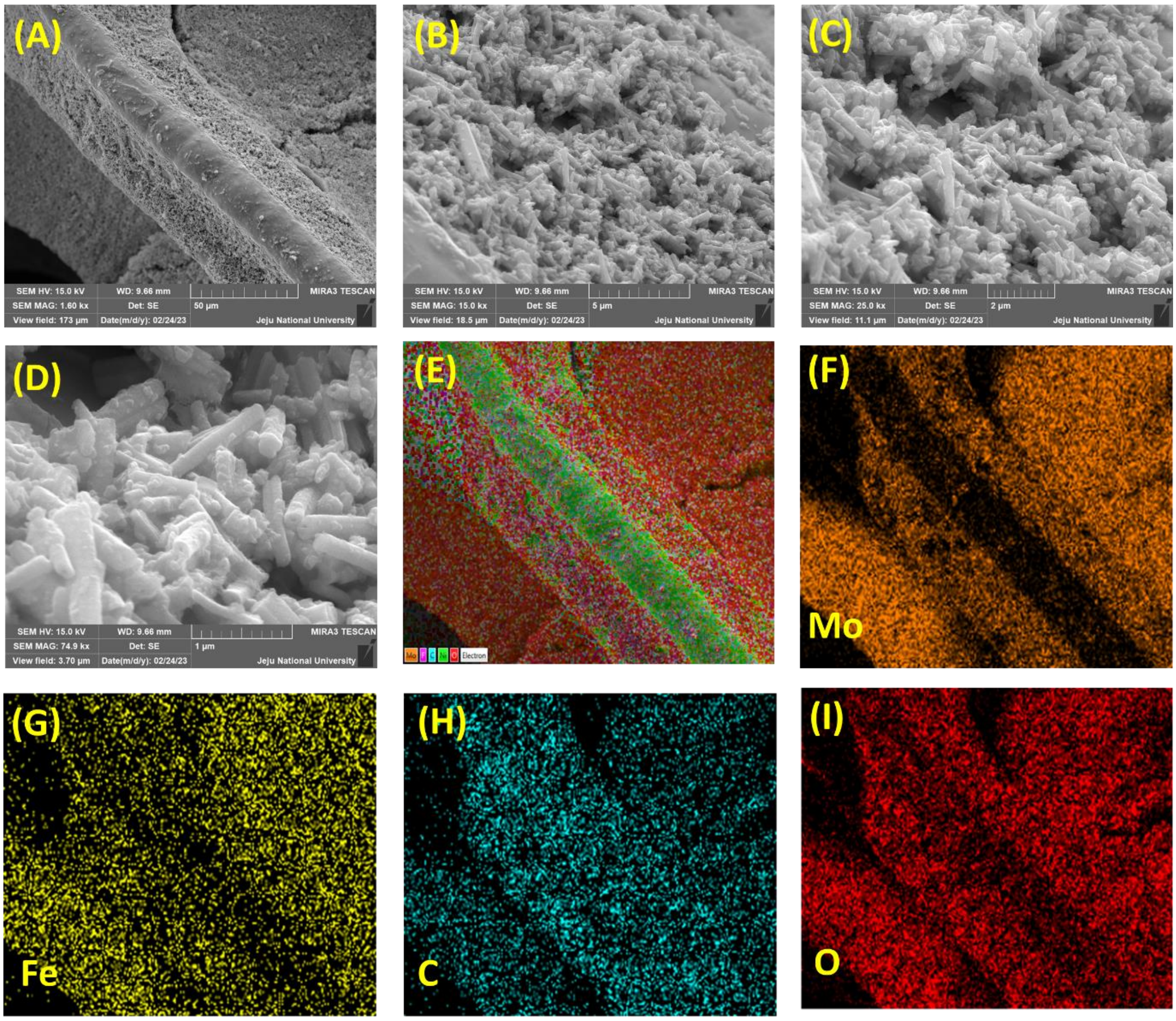

Figure S11. FE-SEM and elemental analysis of as prepared {$Mo_{72}Fe_{30}$} POM electrode. (A-D) Field-emission scanning electron micrographs obtained under different magnifications, (E) overlay image, (F-I) Elemental maps of (F) molybdenum (Mo), (G) iron (Fe), (H) carbon (C), and (I) oxygen (O) respectively.

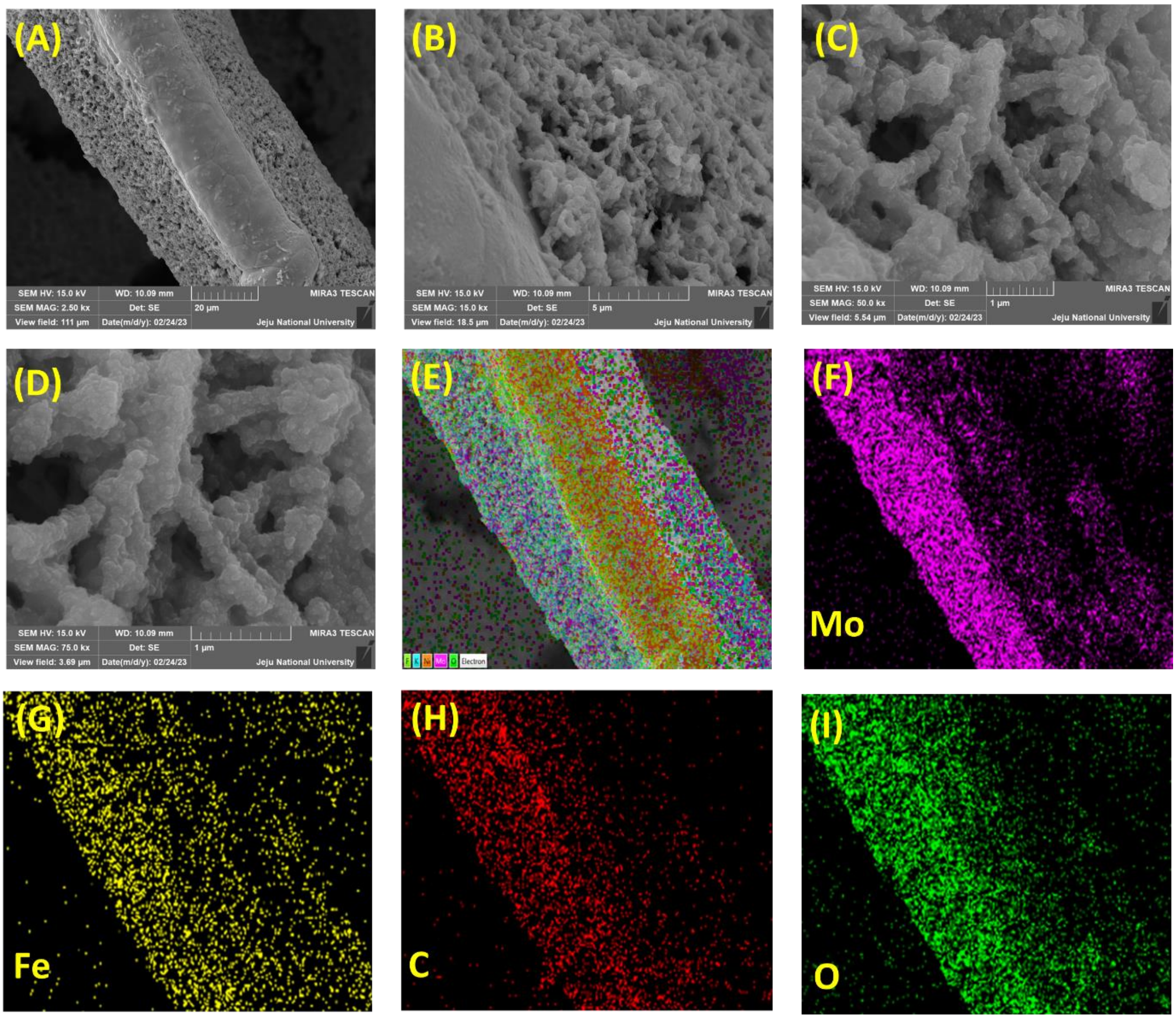

Figure S12. FE-SEM and elemental analysis of {$Mo_{72}Fe_{30}$} POM electrode after electrochemical stability. (A-D) Field-emission scanning electron micrographs obtained under different magnifications, (E) overlay image, (F-I) Elemental maps of (F) molybdenum (Mo), (G) iron (Fe), (H) carbon (C), and (I) oxygen (O) respectively.

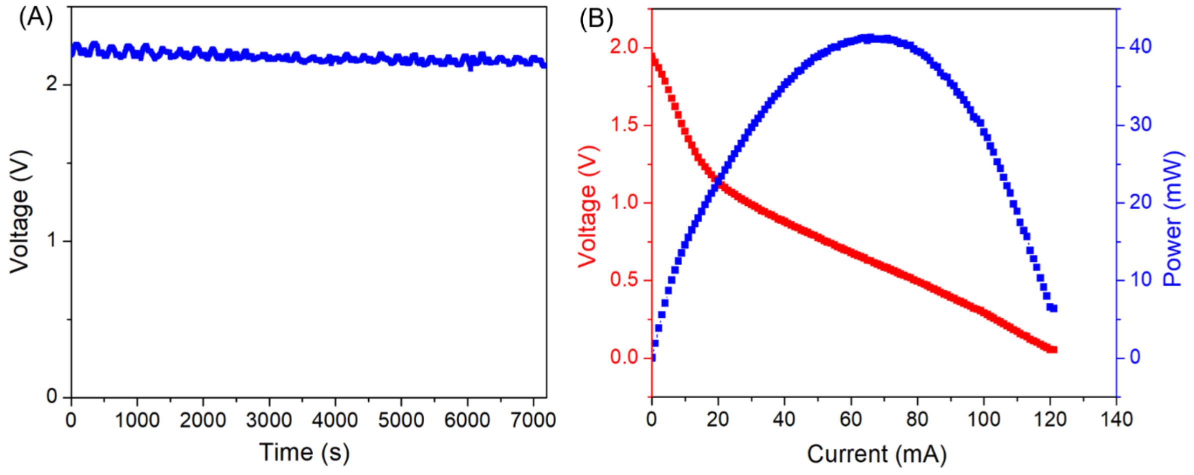

Figure S13. (A) Open-circuit voltage, and (b) Polarization curves of 2.0 V DMFC stack cell measured at room temperature.

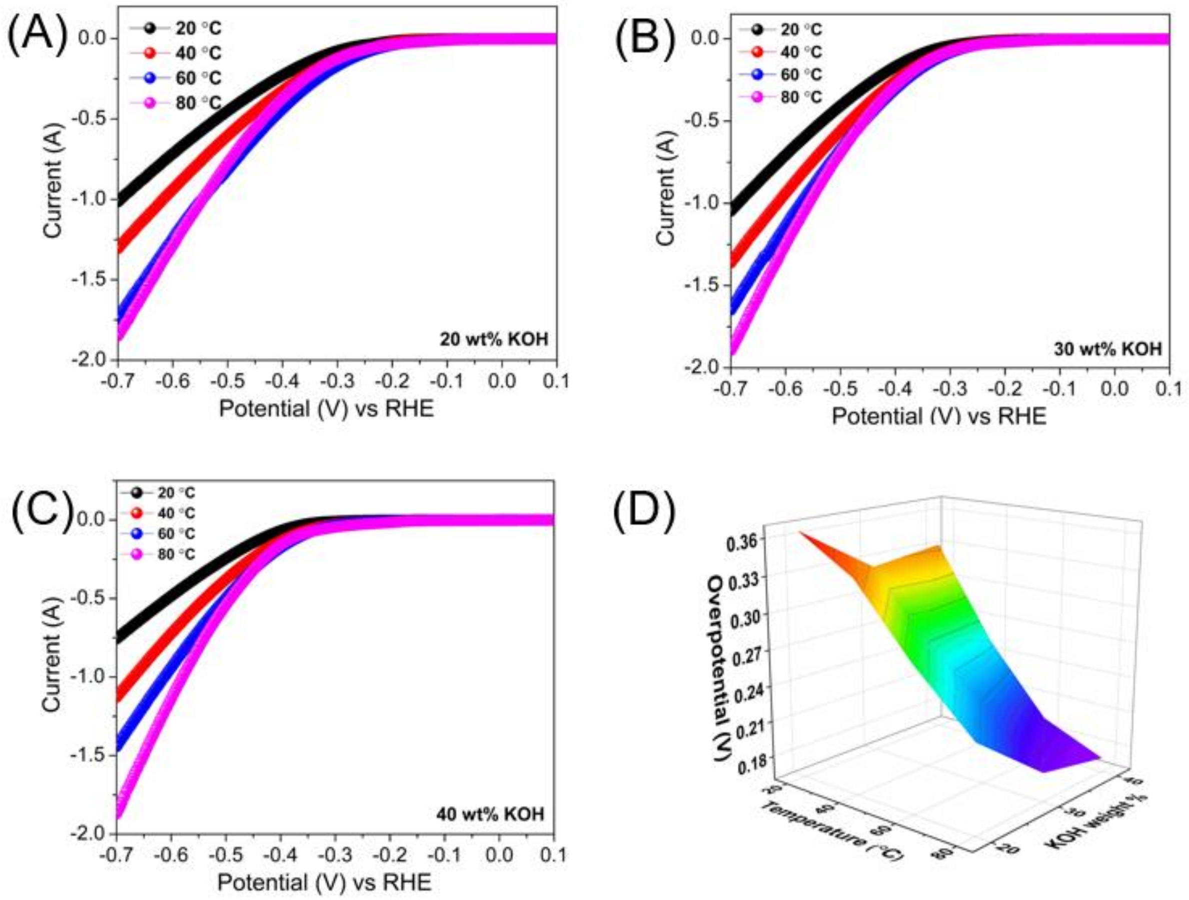

Figure S14. Electrochemical HER analysis of $Mo_{72}Fe_{30}$ POM electrode in various concentrations of KOH electrolyte (20, 30, and 40%) with respect to temperature (20, 40,60, and 80 °C). (A-C) LSV analysis and (D) overpotential value at the current density of -500 mA, respectively.

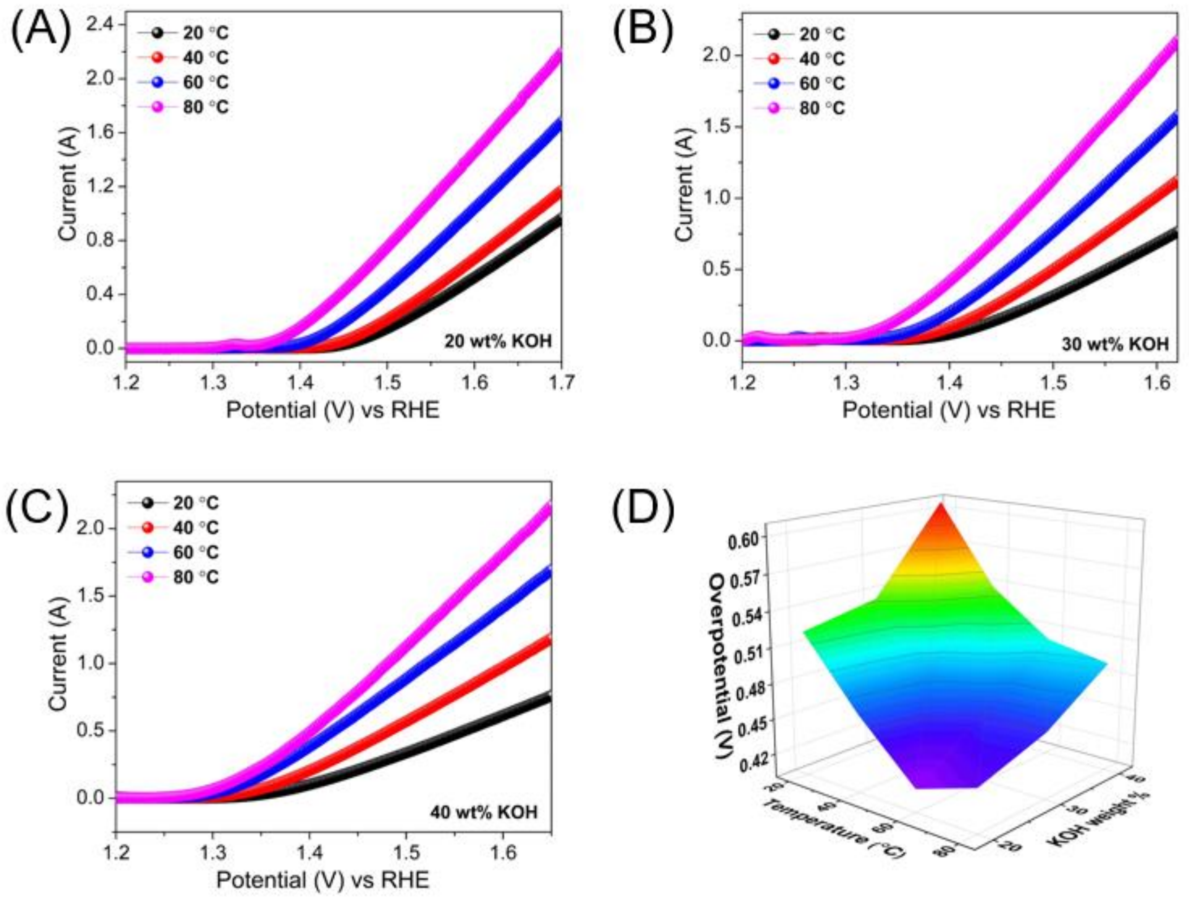

Figure S15. Electrochemical OER analysis of $Mo_{72}Fe_{30}$ POM electrode in various concentrations of KOH electrolyte (20, 30, and 40%) with respect to temperature (20, 40,60, and 80 °C). (A-C) LSV analysis and (D) overpotential value at the current density of 500 mA, respectively.

Table S1: {$Mo_{72}Fe_{30}$} POM electrocatalyst's HER performance comparison over reported HER electrocatalyst in literature.

No.	Catalysts	Electrolyte	Overpotential η (mV) @ 10 mA cm^{-2}	Reference
1.	Ni_2P-Fe_2P/NF	1M KOH	128	[1]
2.	Cr-doped FeNi–P/NCN	1M KOH	190	[2]
3.	FeNi–P/NCN	1M KOH	334	[2]
4.	Nickel–Cobalt–Titanium Alloy	1M KOH	125	[3]
5.	NiCoP/rGO	1M KOH	209	[4]
6.	CuS	1M KOH	181	[5]
7.	FeS_2	1M KOH	255	[5]
8.	$NiCo_2S_4$/Ni	1M KOH	240	[6]
9.	MoS_2/TiN	1M KOH	232 mV	[7]
**10.**	**{$Mo_{72}Fe_{30}$} POM**	**1M KOH**	**135 mV**	**This work**

Table S2: $\{Mo_{72}Fe_{30}\}$ POM electrocatalyst's OER performance comparison over reported OER electrocatalyst in literature.

No.	Catalysts	Electrolyte	Overpotential η (mV) @ *10* mA cm^{-2}	Reference
1.	NiMoN-550	1M KOH	295	[8]
2.	VN-Co-P	1M KOH	335	[9]
3.	$CoMn_2O_4$	1M KOH	310	[10]
4	Fe_{1}-$xCo_{x}[Fe(CN)_{6}]_{0.67}$	1M KOH	460	[11]
5	$Co_3O_4/MnCO_3$	1M KOH	273	[12]
6.	FeOOH	1M KOH	307	[13]
7.	H-NiCo	1M KOH	342	[13]
8.	$FeNi_3$/NiFeOx	1M KOH	285	[14]
9.	FeS_2	1M KOH	330	[5]
**10.**	**$\{Mo_{72}Fe_{30}\}$ POM**	**1M KOH**	**264 mV**	**This work**

Table S3: Comparison on the performance of {$Mo_{72}Fe_{30}$} POM water electrolyzer to that of reported ones in literature.

No.	Catalysts	Electrolyte	Cell Voltage (V) @ *10* mA cm^{-2}	Reference
1.	FeCoSe@NCNSs	1M KOH	1.66 V	[15]
2.	$Co_{0.17}Fe_{0.79}P$/NC	1M KOH	1.66 V	[16]
3.	FeCoNi	1M KOH	1.68 V	[17]
4	CoMnCH	1M KOH	1.68 V	[18]
5	$Co_2P/Mo_2C/Mo_3Co_3C$@C	1M KOH	1.74 V	[19]
6.	CoP/rGO-400	1M KOH	1.70 V	[20]
7.	H-CoP@NC	1M KOH	1.72 V	[21]
8.	Carbon coated Ni_3S_2/MoS_2	1M KOH	1.76 V	[22]
9.	BCNONF	1M KOH	1.79 V	[23]
**10.**	**{$Mo_{72}Fe_{30}$} POM**	**1M KOH**	**1.62 V**	**This work**

References:

[1] L. Wu, L. Yu, F. Zhang, B. McElhenny, D. Luo, A. Karim, S. Chen, Z. Ren, *Adv. Funct. Mater.* **2021**, *31*, 2006484.

[2] Y. Wu, X. Tao, Y. Qing, H. Xu, F. Yang, S. Luo, C. Tian, M. Liu, X. Lu, *Adv. Mater.* **2019**, *31*, 1900178.

[3] P. Ganesan, A. Sivanantham, S. Shanmugam, *ACS Appl. Mater. Interfaces* **2017**, *9*, 12416.

[4] J. Li, M. Yan, X. Zhou, Z.-Q. Huang, Z. Xia, C.-R. Chang, Y. Ma, Y. Qu, *Adv. Funct. Mater.* **2016**, *26*, 6785.

[5] A. Sathyaseelan, D. Kesavan, S. Manoharan, V. K. Mariappan, K. Krishnamoorthy, S.-J. Kim, *ACS Appl. Energy Mater.* **2021**, *4*, 7020.

[6] A. Sivanantham, P. Ganesan, S. Shanmugam, *Adv. Funct. Mater.* **2016**, *26*, 4661.

[7] V. H. Hoa, D. T. Tran, S. Prabhakaran, D. H. Kim, N. Hameed, H. Wang, N. H. Kim, J. H. Lee, *Nano Energy* **2021**, *88*, 106277.

[8] Z. Yin, Y. Sun, C. Zhu, C. Li, X. Zhang, Y. Chen, *J. Mater. Chem. A* **2017**, *5*, 13648.

[9] H. Yang, Y. Hu, D. Huang, T. Xiong, M. Li, M.-S. Balogun, Y. Tong, *Mater. Today Chem.* **2019**, *11*, 1.

[10] A. Bahadur, W. Hussain, S. Iqbal, F. Ullah, M. Shoaib, G. Liu, K. Feng, *J. Mater. Chem. A* **2021**, *9*, 12255.

[11] M. Ishizaki, H. Fujii, K. Toshima, H. Tanno, H. Sutoh, M. Kurihara, *Inorganica Chim. Acta* **2020**, *502*, 119345.

[12] G. Yang, B. Zhu, D. Gao, Y. Fu, J. Zhao, J. Li, *CrystEngComm* **2020**, *22*, 3984.

[13] Y. Shao, M. Zheng, M. Cai, L. He, C. Xu, *Electrochim. Acta* **2017**, *257*, 1.

[14] X. Yan, L. Tian, K. Li, S. Atkins, H. Zhao, J. Murowchick, L. Liu, X. Chen, *Adv. Mater. Interfaces* **2016**, *3*, 1600368.

[15] Y. Pan, M. Wang, M. Li, G. Sun, Y. Chen, Y. Liu, W. Zhu, B. Wang, *J. Energy Chem.* **2021**, DOI 10.1016/j.jechem.2021.12.008.

[16] J. Chen, Y. Zhang, H. Ye, J.-Q. Xie, Y. Li, C. Yan, R. Sun, C.-P. Wong, *ACS Appl. Energy Mater.* **2019**, *2*, 2734.

[17] Y. Yang, Z. Lin, S. Gao, J. Su, Z. Lun, G. Xia, J. Chen, R. Zhang, Q. Chen, *ACS Catal.* **2017**, *7*, 469.

[18] T. Tang, W.-J. Jiang, S. Niu, N. Liu, H. Luo, Y.-Y. Chen, S.-F. Jin, F. Gao, L.-J. Wan, J.-S. Hu, *J. Am. Chem. Soc.* **2017**, *139*, 8320.

[19] X. Li, X. Wang, J. Zhou, L. Han, C. Sun, Q. Wang, Z. Su, *J. Mater. Chem. A* **2018**, *6*,

5789.

[20] L. Jiao, Y.-X. Zhou, H.-L. Jiang, *Chem. Sci.* **2016**, *7*, 1690.

[21] Y. Xie, M. Chen, M. Cai, J. Teng, H. Huang, Y. Fan, M. Barboiu, D. Wang, C.-Y. Su, *Inorg. Chem.* **2019**, *58*, 14652.

[22] C. Wang, L. Kong, H. Sun, M. Zhong, H. Cui, Y. Zhang, D. Wang, J. Zhu, X. Bu, *ChemElectroChem* **2019**, *6*, 5603.

[23] H. Li, B. Ren, W. Liu, L. Jing, R. Y. Tay, S. H. Tsang, L. Ricardez–Sandoval, A. Yu, E. H. T. Teo, *Nano Energy* **2021**, *88*, 106246.
